# Persönliche Präferenz, Erfahrung, Intuition und operative Schule dominieren die Verwendung postoperativer Wunddrainagen in der Dermatochirurgie

**DOI:** 10.1007/s00105-020-04709-w

**Published:** 2020-10-28

**Authors:** Stephanie Sophia Ruers, Stefan Wagenpfeil, Gerd Gauglitz, Moritz Felcht, Tino Wetzig, Falk G. Bechara, Wolfgang Koenen, Christian Kunte, Guido Bruning, Cornelia S. L. Müller

**Affiliations:** 1 Klinik für Dermatologie, Venerologie und Allergologie, Universitätsklinikum des Saarlandes, Campus Homburg, Homburg, Deutschland; 2 Institut für Medizinische Biometrie, Epidemiologie und Medizinische Informatik, Campus Homburg, Universität des Saarlandes, Homburg, Deutschland; 3 Klinik und Poliklinik für Dermatologie und Allergologie, Ludwig-Maximilians-Universität München, München, Deutschland; 4 Universitätsmedizin Mannheim, Medizinische Fakultät Mannheim, Ruprecht-Karls-Universität Heidelberg, Mannheim, Deutschland; 5 Klinik für Dermatologie, Dermatochirurgie und Allergologie, Asklepios Klinik Weißenfels, Weißenfels, Deutschland; 6 Klinik für Dermatologie, Venerologie und Allergologie, St. Josef Hospital, Ruhr-Universität Bochum, Bochum, Deutschland; 7 Klinik für Dermatologie und Venerologie, Dermatologie im Fronhof, Bad Dürkheim, Deutschland; 8 Abteilung Dermatochirurgie und Dermatologie, Artemed Fachklinik München, München, Deutschland; 9 Tabea GmbH & Co.KG, Kösterbergstr. 32, Hamburg, Deutschland

**Keywords:** Behandlungsverhalten, Indikationsstellung, Sicherheitsbedürfnis, Eminenzbasiertes Handeln, Postoperativer Verlauf, Treatment approach, Indications, Safety needs, Eminence-based actions, Postoperative course

## Abstract

**Hintergrund:**

Die Verwendung von Drainagesystemen in der Dermatochirurgie erfolgt bislang ohne evidenzbasierte Daten. Indikationen, Komplikationen und Kontraindikationen werden traditionell von Operateur zu Operateur weitergegeben, sind jedoch bisher nicht definiert.

**Methodik:**

Es wurde eine internetbasierte Umfrage erstellt und unter den Mitgliedern der DGDC e. V. (Deutsche Gesellschaft für Dermatochirurgie e. V.) ausgesandt. Abgefragt wurden das allgemeine Behandlungsverhalten im deutschsprachigen Raum in Bezug auf die Anwendung der Wunddrainage nach dermatologischen Operationen sowie die Nutzungsgewohnheiten und Erfahrungen der Kollegen mit Drainage-assoziierten Komplikationen.

**Ergebnisse:**

Es haben 12,73 % der angeschriebenen DGDC-Mitglieder den Fragebogen beantwortet. Drainagen werden überwiegend im klinischen Umfeld eingesetzt, es werden alle abgefragten Drainagesysteme verwendet. Ausmaß und Komplexität des Eingriffs sind die wesentlichen Kriterien bei der Indikationsstellung. Der Einsatz von Drainagen ist abhängig vom Alter des Teilnehmers und erfolgt mehrheitlich bei Patienten, bei denen Komplikationen im postoperativen Verlauf erwartet werden (Adipositas, Nikotinabusus, Diabetiker).

**Diskussion:**

Zusammenfassend verwendet die Mehrzahl der Teilnehmer Wunddrainagen und dies mehrheitlich intuitiv. Einheitliche fixe evidenzbasierte Parameter rund um die Verwendung von Wunddrainagen fehlen. Bei der Beurteilung der Notwendigkeit einer Wunddrainage scheint ein individuell unterschiedlich ausgeprägtes Sicherheitsbedürfnis bei den einen und „eminenzbasiertes“ Handeln bei den anderen Dermatochirurgen eine große Rolle zu spielen.

## Abstract (en)

**Background:**

The use of drainage systems in dermatosurgery has so far been carried out without evidence-based data. The indications, complications and contraindications are traditionally passed on from surgeon to surgeon but have so far not been defined.

**Method:**

An Internet-based survey was created and sent out to members of the German Society for Dermatosurgery (DGDC). The questions were on the general treatment approach in German language countries with reference to the use of wound drainage following dermatological operations as well as the utilization habits and experiences with drainage-associated complications.

**Results:**

Of the DGDC members contacted 12.73% completed the questionnaire. Drainages were predominantly used in the clinical environment and all drainage systems in question were used. The extent and complexity of the intervention were essential criteria when evaluating the indications. The use of drainages was dependent on the age of the participant and mostly carried out in patients where complications in the postoperative course were to be expected (e.g. obesity, nicotine use, diabetes).

**Conclusion:**

In summary, the majority of the participants used wound drainages and mostly intuitively. Uniform and fixed evidence-based parameters for the use of wound drainages are lacking. In the assessment of the necessity for a wound drainage, an individually expressed need of safety seems to play a large role for some dermatosurgeons and an eminence-based action for others.

Wunddrainagen werden seit vielen Jahren in allen chirurgischen Fächern eingesetzt. Traditionelle Ziele der Wunddrainage umfassen die Ableitung von Blut und Wundsekret aus der Wundhöhle im Anschluss an eine Operation, Reduktion postoperativer Hämatome und Wundinfektionen. Zusätzlich sollen die Wundränder adaptiert und stabilisiert werden. J.O. Robinson aus dem Jahr 1986 und J.M. Meyerson 2015 thematisieren ausführlich sämtliche geschichtliche und technische Aspekte von Wunddrainagen [1, 2]. Während vergleichsweise viele Untersuchungen und Berichte zum Einsatz von Wunddrainagen in der Abdominalchirurgie, plastischen Chirurgie, Gynäkologie und auch im orthopädisch-unfallchirurgischen Sektor vorliegen [3–5], so ist die Evidenz im dermatochirurgischen Sektor diesbezüglich mehr als spärlich. Die Evidenzlage für einen Nutzen ist allerdings auch in anderen Fachgebieten kümmerlich und unter Debatte, es liegen aber mehr Arbeiten zur Thematik vor. Insgesamt werden der Nutzen und damit die Erfordernis von Wunddrainagen in der Dermatochirurgie, in anderen chirurgischen Fachdisziplinen und auch in der Literatur bei mangelhafter Evidenzfrage kontrovers debattiert [6, 7].

Die Kommission für Krankenhaushygiene und Infektionsprävention des Robert Koch-Instituts hat bereits 2007 folgende Empfehlungen zur Verwendung von Wunddrainagen publiziert [8]:

1. „Wunddrainagen sollen nicht routinemäßig, sondern nur bei klarer Indikation und so kurzzeitig wie möglich eingesetzt werden.“
2. „Offene Drainagen sollen aus infektionspräventiven Gründen vermieden werden.“
3. „Drainagen sollen nicht über die OP-Wunde, sondern über eine separate Inzision gelegt werden.“

Für den dermatochirurgischen Indikationsbereich sind nahezu keine validen Daten hinsichtlich des Indikationsspektrums, der Behandlungsmodalitäten oder assoziierter Komplikationen verfügbar. Die Verwendung von Drainagesystemen in der Dermatochirurgie ist daher wesentlich von persönlichen Präferenzen der Anwender sowie „chirurgischer Schule“ abhängig. Im Jahr 2019 konnte unsere Arbeitsgruppe erstmals in einer retrospektiven Arbeit unsere eigenen Erfahrungen mit Wunddrainagen an einem recht großen Patientenkollektiv dermatochirurgischer Patienten beschreiben [9].

Ziel dieser Umfrage unter den Mitgliedern der DGDC e. V. (Deutsche Gesellschaft für Dermatochirurgie e. V.) als repräsentative Gruppe dermatologischer Chirurgen war es, das allgemeine Behandlungsverhalten im deutschsprachigen Raum in Bezug auf die Anwendung von Wunddrainagen nach dermatologischen Operationen zu untersuchen sowie die Nutzungsgewohnheiten und Erfahrungen der Kollegen mit Drainage-assoziierten Komplikationen in diesem Beispielkollektiv zu erfassen.

## Methodik

Es wurde eine internetbasierte Umfrage mit insgesamt 17 Fragen zur Verwendung von Wunddrainagen erstellt (Tab. 1). Diese Online-Umfrage wurde an alle Mitglieder der Deutschen Gesellschaft für Dermatochirurgie (DGDC e. V.) ausgesandt, von denen zum Stichtag 14.09.2017 eine E‑Mail-Adresse vorlag (*n* = 965). Die Aussendung der Online-Umfrage erfolgte einmalig. Es erfolgten weder Erinnerungsmails noch wurden Anreize (beispielsweise Gutscheinverlosung oder Werbegeschenke) zur Steigerung der Beantwortungsquote ausgelobt. Jeder Teilnehmer konnte durch Anwendung einer Protokolldatei gegen Mehrfachabstimmung nur einmalig an der Umfrage teilnehmen. Im Rahmen der Umfrage wurden keine Personendaten erhoben; die Umfrage wurde somit vollständig anonym erhoben und ausgewertet. Außer den Fragen zu Alter und Geschlecht waren alle Fragen freiwillig und konnten auch unbeantwortet bleiben.

**Table 1:** 

Geschlecht		
Alter		
Ihre Einrichtung: Klinik/Praxis		
	*Frage*	*Antwortmöglichkeiten*
1	Wieviele Eingriffe tätigen Sie pro Jahr?	Offene Frage
2	Verwenden Sie Wunddrainagen in Ihrer Einrichtung?	□ Ja □ Nein □ falls ja, weiter mit Frage 3; 2.1 falls nein, warum nicht?
3	Welche Wirkung erwarten Sie von einer Wunddrainage?	Offene Frage
4	Welche Drainagesysteme verwenden Sie? (Mehrfachantwort möglich)	□ Niedervakuum □ Hochvakuum □ Redon-Drainage □ Spül-Saug-Drainage □ Lasche □ andere 4.1. falls andere, welche?
5	Von welchen Faktoren machen Sie die Verwendung einer Wunddrainage abhängig? (Mehrfachantwort möglich)	□ Größe der Operation □ Dauer der Operation □ Komplexer Wundverschluss (Lappenplastiken o. ä.) □ Andere 5.1. falls andere, welche?
6	Bei welchen Operationen verwenden Sie häufig Wunddrainagen?	Offene Frage
7	Am wievielten Tag entfernen Sie die Drainage?	□ Am 1. postoperativen Tag? □ Am 2. postoperativen Tag? □ Am 3. postoperativen Tag? □ Nach mehr als drei Tagen □ Keine feste Vorgabe
8	Machen Sie das Entfernen der Drainage von der geförderten Exsudatmenge abhängig?	□ Nein □ Ja Wenn ja, ab welcher Fördermenge?
9	Haben Sie Komplikationen im Zusammenhang mit der Verwendung von Wunddrainagen bemerkt? (Mehrfachantwort möglich)	□ Nein, keine □ Blutungen □ Wundinfektionen □ Schmerzen □ Lymphfistel □ Technische Probleme mit der Drainage (z. B. Undichtigkeit etc.) □ Andere 9.1. wenn andere, welche?
10	Konnten Sie Komplikationen vermehrt in einer der folgenden Patientengruppen beobachten? (Mehrfachantwort möglich)	□ Nein □ Ältere Patienten (>60. Lebensjahr) □ Männer □ Frauen □ Diabetiker □ Adipositas. Patienten □ Raucher □ Patienten mit Gefäßerkrankungen (CVI, pAvK o. ä.) □ andere 10.1 falls andere, welche?
11	Konnten Sie vermehrt postoperative Wundinfektionen beobachten, wenn eine Wunddrainage verwendet wurde?	□ Nein, keine □ Ja 11.1 Wenn ja, welche?
12	Verwenden Sie bei bestimmten Patientengruppen per se häufiger eine Wunddrainage? (Mehrfachantwort möglich)	□ Nein, bei keiner Gruppe □ Ältere Patienten (>60. Lebensjahr) □ Männer □ Frauen □ Diabetiker □ Adipositas. Patienten □ Raucher □ Patienten mit Gefäßerkrankungen (CVI, pAvK o. ä.) □ andere 12.1 falls andere, welche?
13	Verzichten Sie bei bestimmten Vorerkrankungen auf das Legen von Wunddrainagen?	□ Nein □ Ja 13.1 Wenn ja, bei welchen?
14	Haben Sie den Eindruck, dass Wunddrainagen den Heilungsprozess manchmal behindern?	□ Nein □ Ja 14.1 Wenn ja, wie ist Ihr Eindruck?
15	Geben Sie Ihren Patienten mit einer Wunddrainage auch eine postoperative Antibiose?	□ Nein □ Ja 15.1 Wenn ja, in welchen Fällen?
16	Verzichten Sie heute bei Operationen auf Wunddrainagen, bei denen Sie früher solche verwendet hätten?	□ Nein □ Ja 16.1 Wenn ja, warum?
17	Beachten Sie Kontraindikationen hinsichtlich der Verwendung von Wunddrainagen?	□ Nein □ Ja 17.1 Welche Kontraindikationen beachten Sie??

Von technischer Seite wurden die Erstellung sowie der Versand der Online-Umfrage durch die Firma Transparent Werbeagentur GbR, Chemnitz, betreut. Basis für den Online-Fragebogen war ein Formular im Content-Management-System Contao. Die ausgefüllten Fragebögen („Antwortmails“) wurden direkt an die Leiterin der Studie (Letztautorin, CM) gesandt. Zugriff auf diese Antwortmails hatten ausschließlich die Erst- und Seniorautorin (SR und CM) dieser Studie.

Die statistische Analyse und grafische Darstellung der erhobenen Daten erfolgten mittels Tabellenkalkulation durch das Programm Excel der Firma Microsoft® (Version 12.0; Redmond, WA 98052-6399 USA) sowie IBM-SPSS Statistics Version 24 (IBM SPSS, Ehningen). Für Vergleiche von relativen Häufigkeiten wurde je nach Adäquatheit der χ^2^-Test bzw. der exakte Test von Fisher verwendet. Da begründete Vermutungen anhand einer breit gefächerten Literaturrecherche sowie logisch argumentativen Schlüssen vorlagen, wurde der exakte Test von Fisher einseitig durchgeführt. Das Signifikanzniveau betrug 5 %. *p*-Werte, die kleiner als 0,05 waren, wurden somit als statistisch signifikant erachtet. Wegen des explorativen Charakters der Studie erfolgte keine Adjustierung für multiple Tests. Die offenen Fragen des Fragebogens ließen häufig aufgrund der Verschiedenheit der gegebenen Antworten keine Bildung von Sammelbegriffen zu. Daher konnten diese Fragen statistisch nicht ausgewertet werden, sondern wurden einzeln aufgelistet und diskutiert.

## Ergebnisse

### Allgemeine Aspekte der Verwendung von Wunddrainagen

**Figure 1:**
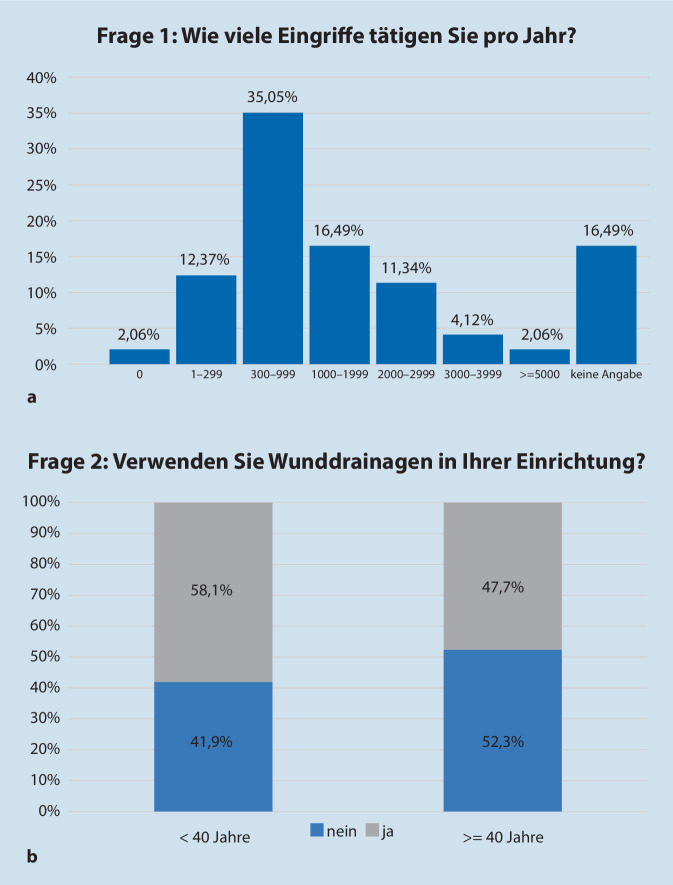

Zum Stichtag der Aussendung der Online-Umfrage hatte die DGDC e. V. 1094 Mitglieder (574/1094, 52,4 % Männer, 520/1094, 47,5 % Frauen); bei 965/1094 (88,2 %) lag eine funktionierende E‑Mail-Adresse vor. Von 203/1094 angeschriebenen E‑Mail-Adressen wurde eine Nichtzustellungsbenachrichtigung erzeugt, sodass davon auszugehen ist, dass 762/1094 Mitglieder der DGDC e. V. effektiv erreicht wurden; 97/762 Mitglieder haben die Umfrage beantwortet. Dies entspricht einer Rücklaufquote von 12,73 %. Unter den Teilnehmern waren 42/97, 42,3 % Männer und 55/97, 56,7 % Frauen; 57,7 % der Teilnehmer sind in der Klinik tätig; 40,2 % sind in Praxen/Niederlassung tätig. Gut ein Drittel der Teilnehmer gab an, zwischen 40 und 49 Jahre alt zu sein; 24 % sind zwischen 30 und 39 Jahre alt und weitere 25 % 50 bis 59 Jahre alt; 9 % der Teilnehmer waren älter als 60 Jahre; 35 % der Teilnehmer gaben an, zwischen 300 und 999 operative Eingriffe/Jahr durchzuführen (Abb. 1a).

Generell gaben 78,6 % der in Kliniken tätigen Teilnehmer an, Wunddrainagen einzusetzen, während nur 12,8 % der niedergelassenen Kollegen dies angaben. Dieser Unterschied war statistisch signifikant (*p* < 0,001).

In Tab. 2 sind die Antworten der Teilnehmer auf die Frage 2.1 aufgeführt, aus welchen Gründen sie keine Wunddrainagen verwenden. Teilnehmer, die Frage 2.1 ausgefüllt haben, haben die Umfrage an dieser Stelle beendet.

**Table 2:** 

„Keine Durchführung von großen chirurgischen Operationen in dieser Praxis, die die Verwendung von Wunddrainagen notwendig machten“
„Nicht nötig“; „Zu kleine Eingriffe“; „Keine Indikation bei kleineren Eingriffen“
„Zumeist Hautexzisionen mit Primärverschluss, die keiner Drainage bedürfen“
„Ambulante Operationen fallen nicht so groß aus“
„Nur bei stationären Patienten“
„Nicht erforderlich bei sorgfältiger Präparation“
„Halte ich für aufwendig und ohne wirklichen Nutzen“
„Industriegesponserter Mist“
„Durch gute postoperative Kompression entfällt die Indikation zur Wunddrainage“

Generell verwenden 24,5 % der Teilnehmer Hochvakuumdrainagen und 31 % Niedervakuumdrainagen; 89,8 % aller Teilnehmer verwenden Redondrainagen, 67,4 % Laschendrainagen, und nur 28,6 % verwenden auch komplexe Spül-Saug-Drainagen. Das Spektrum der verschiedenen Drainagesysteme variiert zwischen den Anwendern in Kliniken und Praxen erheblich: In Kliniken werden bis zu 5 verschiedene Systeme verwendet: 35,7 % der Teilnehmer aus den Kliniken gaben an, regelhaft genau 3 verschiedene Drainagesysteme einzusetzen, 26 % verwenden 2 verschiedene Drainagesysteme. In den Praxen werden hingegen von je 40 % der Teilnehmer ausschließlich 1 oder 2 verschiedene Drainagesysteme verwendet. Unterschiede auf Signifikanzniveau zwischen Teilnehmern aus Kliniken und Praxen hinsichtlich der verschiedenen Drainagesysteme konnten nur in der Verwendung von Redondrainagen beobachtet werden: Diese werden von 100 % der Teilnehmer aus Kliniken verwendet und nur von 40 % der Praxen (*p* < 0,001). Tendenziell werden in Kliniken mehr Saug-Spül-Drainagen sowie Hochvakuumdrainagen eingesetzt, während 100 % der Teilnehmer aus den Praxen Laschendrainagen verwenden.

Die durch Verwendung von Wunddrainagen erhofften Effekte sind in Tab. 3 aufgelistet und umfassen mehrheitlich den Wunsch, Hämatome und Serome zu vermeiden, eine bessere und schnellere Wundheilung zu gewährleisten, weniger Wundinfektionen zu generieren und eine Abflusserleichterung bei Nachblutungen zu erzielen.

**Table 3:** 

„Vermeidung eines Seroms/Hämatoms, bessere Wundheilung speziell bei Lappenplastiken“
„Geringeres Risiko einer Hämatombildung, *weniger Wundinfekte*“
„Schnellere und komplikationsärmere Wundheilung“
„Wundsekretion ableiten, evtl. Sickerblutungen ableiten“
„*Vermeidung von Wundinfektionen*“
„Verminderung postoperativer Komplikationen“
„Schnellere Heilung“
„Bessere Wundheilung, frühere Feststellbarkeit einer Nachblutung“
„Förderung der Wundheilung durch den Sog“
„Vakuumsystem führt Wundränder zusammen“
„Ableitung von Tumeszenzanästhesie“
„*Infektionsprophylaxe*“
„*Weniger postoperative Wundinfektionen*“

In Kursiv wurden Schlagworte dargestellt, die geantwortet wurden, zur besseren Visualisierung der diversen offenen Antworten.

### Indikationen und Faktoren, die zur Nutzung einer Wunddrainage bewegen

Die wichtigsten Faktoren, die durch die Teilnehmer als Indikation zu einer Wunddrainage angeben werden, sind die Operationsgröße als Ausmaß des Eingriffs (81,6 %) sowie komplexe Wundverschlüsse (75,5 %). Hingegen spielt die Operationsdauer nur bei 22,5 % der Teilnehmer eine entscheidende Rolle. Als weitere Gründe zur Anlage einer Drainage wurden von 55,6 % der Teilnehmer „Größe, Tiefe und Lokalisation der Wunde“ sowie von 33,3 % „blutungsassoziierte Gründe“ angegeben. Individuell wurden unter der Rubrik „Sonstiges“ in Form freier Antworten die in Tab. 4 genannten Indikationen angegeben.

**Table 4:** 

„Großflächige Verschiebe- und Rotationslappenplastiken, mikrovaskulär anastomosierte Lappen“
„Sentinellymphknotenbiopsie, bei großen Lappenplastiken“
„Abszessspaltungen“
„Varizenstripping“
„Lipomoperation“
„Acne inversa“
„Hyperhidrose“
„Exzisionen von subkutan gelegenen Prozessen“
„Bei starken intraoperativen Blutungen“
„Schweißdrüsenkürettage“
„Transpositionslappenplastiken“
„Infizierte Wunden“
„Patienten unter Antikoagulation“

### Drainageverweildauer

**Figure 2:**
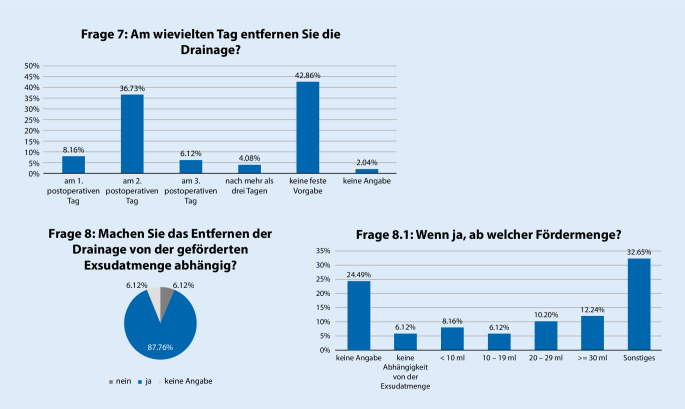

Es gaben 42,9 % der Teilnehmer an, keine fixe Vorgabe zu befolgen, wann die Drainage postoperativ entfernt wird; 36,7 % der Teilnehmer gaben jedoch an, die Drainage am 2. postoperativen Tag zu entfernen, wobei 87,8 % der Teilnehmer die Entfernung der Drainage von der geförderten Exsudatmenge abhängig machen (Abb. 2).

### Komplikationen im Zusammenhang mit Wunddrainagen

**Figure 3:**
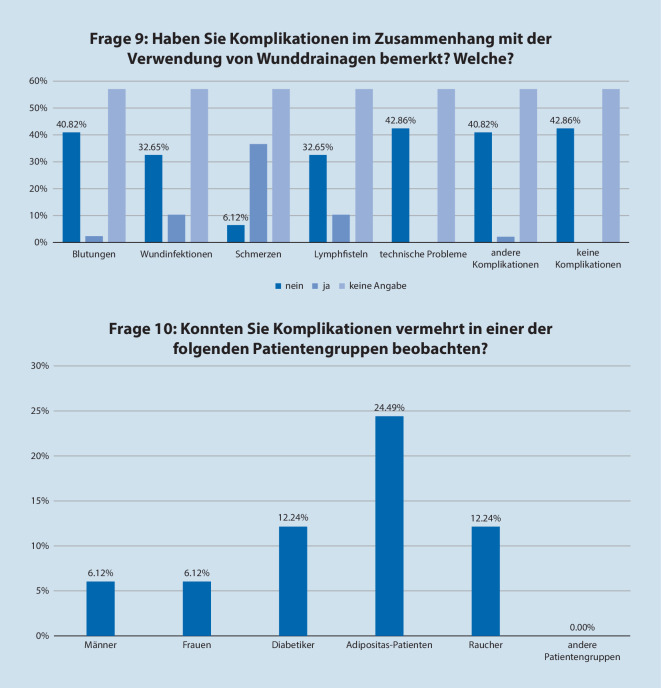

Es gaben 57 % der Teilnehmer an, keine Drainage-assoziierten Komplikationen bemerkt zu haben. Unter den in Frage 9 angebotenen Komplikationen gaben 36,7 % Schmerzen, je 10,2 % Wundinfektionen und Lymphfisteln und 2 % Blutungen an. Hierbei waren es bei einem Viertel der Befragten Adipositaspatienten und zu je 12,2 % Diabetiker und Raucher, bei denen die oben genannten Komplikationen beobachtet wurden (Abb. 3). Das Geschlecht der Patienten schien bei keinem der Teilnehmer hinsichtlich des Auftretens von Komplikationen eine Rolle zu spielen. Folgerichtig gaben 16,3 % der Teilnehmer an, bei Patienten mit Adipositas per se häufiger eine Wunddrainage zu verwenden, gefolgt von 4 % bei Diabetikern. Bei Rauchern gab kein Teilnehmer an, per se häufiger eine Wunddrainage zu nutzen.

Nur 4 % der Teilnehmer hatten den Eindruck, dass Wunddrainagen den Heilungsprozess behindern können, geben dafür jedoch keine Gründe an; 16,3 % der Teilnehmer geben an, generell bei Verwendung von Drainagen auch eine postoperative Antibiotikabehandlung durchzuführen. Gründe hierfür sind in Tab. 5 aufgelistet.

**Table 5:** 

„Bei Plastiken“
„Ulcus cruris“
„Unterschiedliche Indikationen“
„Komplizierte operative Verhältnisse“
„Bei ausgedehnten Operationen oder septischen Eingriffen“
Entscheid nach Kontamination und/oder Dauer der Operation

Es gaben 14,3 % der Teilnehmer an, heutzutage bei diversen Operationen auf Wunddrainagen zu verzichten, bei denen sie früher welche verwendet hätten. Die Gründe hierfür sind in Tab. 6 gezeigt.

**Table 6:** 

„Insgesamt weniger häufig“
„Bessere Blutstillung direkt intraoperativ“
Sentinel-OP in axillärer Lokalisation haben seltener Serome, daher Drainage verzichtbar
„Bei Tumeszenzanästhesie häufig nicht notwendig“; „Unter Tumeszenzanästhesie sind Drainagen kaum noch notwendig, weil es nicht nachblutet“
„Wunddrainagen sind im ambulanten postoperativen Verlauf kompliziert und unbequem für die Patienten“
„Mehr Erfahrung, wann ein Verzicht möglich ist“

Es gaben 18,4 % der Teilnehmer an, Kontraindikationen bei der Verwendung von Drainagesystemen zu beachten. In Tab. 7 sind die genannten Kontraindikationen aufgelistet. Angaben zu Drainage-assoziierten Komplikationen waren weder abhängig vom Geschlecht der Teilnehmer noch von der Institution, in der der Teilnehmer arbeitete (Klinik vs. Praxis).

**Table 7:** 

Nekrosen, Fisteln, maligne Tumoren
Operation in der Nähe von größeren Gefäßen
Schlechte Wundumgebung
Gefäßnähe
Allergien
Unverträglichkeit des Materials

### Altersabhängigkeit der Ergebnisse

Generell nicht signifikant, aber mit einem erkennbaren Trend gaben jüngere Teilnehmer (<40 Jahre) an, generell öfter Drainagen zu verwenden als ältere Teilnehmer (>40 Jahre) (58 % vs. 48 %) (Abb. 1b). Auch die Operationsgröße spielt bei jüngeren Kollegen eine größere Rolle: Teilnehmer <40 Jahre verwenden häufiger Drainagen als ältere (100 % vs. 88 %); dies gilt in gleichem Maße für komplexe Wundverschlüsse (95 % vs. 80 %). Jedoch verwenden ältere Teilnehmer häufiger Drainagen bei längeren Operationen (24 % der >40-Jährigen vs. 27 % der <40-Jährigen).

## Diskussion

Bislang ist die Datenlage zur Anwendung von Wunddrainagen in der Dermatochirurgie nicht ausreichend. Es existieren weder definierte Indikationen noch Kontraindikationen und ebenso keine fixen Parameter, nach wie viel Tagen die Drainage entfernt werden sollte, bzw. keine Angaben zur Exsudatmenge, ab welcher eine Entfernung der Drainage möglich ist. Generell umfasst die Dermatochirurgie operative Eingriffe an der Körperoberfläche bis zu den Faszien, und Wunddrainagen sind nicht die generelle Regel. Eingriffe an Körperhöhlen werden nicht durchgeführt. Die in dieser Umfrage gelegentlich genannten Eingriffe an der Parotis sind wahrscheinlich auf einzelne Mitglieder der DGDC e. V. mit chirurgischer oder anderer Zweitfacharztbezeichnung (z. B. Facharzt für plastische und ästhetische Chirurgie) und/oder der Zusatzweiterbildung plastische Operationen zurückzuführen. Aufgrund der geringen Datenmenge zu Wunddrainagen im dermatochirurgischen Fachbereich wurden die Ergebnisse dieser Umfrage vorwiegend anhand von Drainagen in der Allgemeinchirurgie, Gynäkologie und Orthopädie diskutiert.

Unsere Arbeitsgruppe konnte 2019 erstmalig in einer retrospektiven Analyse an 495 Patienten die Indikationen beschreiben, bei denen an einem dermatochirurgischen Zentrum Drainagen verwendet werden: Hierbei wurden am häufigsten Eingriffe am Lymphgefäßsystem (Sentinellymphknotenbiopsien) genannt, gefolgt von lokalen Lappenplastiken, Dehnungsplastiken und Saugkürettagen. An Komplikationen wurden im Untersuchungszeitraum zwischen Januar 2010 und Dezember 2014 in absteigender Reihenfolge Schwellungen, Rötungen der Wunde, Nachblutung/Hämatombildung, Anstieg der Entzündungsparameter, Lymphfisteln und Nervenfunktionsstörungen beschrieben [9]. Wir konnten zeigen, dass eine statistische Signifikanz zwischen dem Alter der Patienten und dem Auftreten postoperativer Komplikationen, v. a. Schwellungen, zu beobachten ist; wenngleich sich diesbezüglich die Patienten mit und ohne Wunddrainage nicht unterscheiden. Ebenso gab es keine signifikanten Unterschiede zwischen Patienten mit und ohne Wunddrainage hinsichtlich weiterer Faktoren wie Geschlecht, Nikotinabusus, Komorbidität, Antikoagulation und weiteren Faktoren [9].

In der vorliegenden Umfrage konnte herausgearbeitet werden, dass generell in Kliniken tätige Teilnehmer gehäuft Drainagesysteme in der Wundversorgung nach dermatochirurgischen Eingriffen nutzen und ihre Entscheidung für oder gegen eine Drainage mehrheitlich von der Operationsgröße sowie der erwarteten Komplexität des Eingriffes abhängig machen. Dies spiegelt sich auch in den Erfahrungen von Simgen et al. wieder, da auch in diesem Kollektiv Drainagen signifikant öfter bei höheren intraoperativen Defektgrößen eingesetzt wurden [9]. Die Operationsdauer spielte in der aktuellen Umfrage nur bei knapp einem Viertel der Teilnehmer eine Rolle. Ebenso werden lokalisationsspezifische Faktoren von Simgen et al. als auch von den Teilnehmern dieser Studie bei der Entscheidung für oder gegen Wunddrainagen angegeben [9]. Die individuell angegebenen Indikationen zur Wunddrainage reflektieren das doch deutlich differente Spektrum an operativen Eingriffen zwischen Kliniken und Praxen und erklären die nur niedrige Zahl an Kollegen, die aus Praxen heraus Wunddrainagen einsetzen. Generell werden Patienten, die in Praxen operiert werden, nicht mehrtägig stationär nachbehandelt. Somit entfällt auch die Möglichkeit, Wunddrainagesysteme ambulant zu betreuen. Hierdurch und durch die Tatsache, dass ambulant generell kleinere und oberflächlichere Eingriffe durchgeführt werden, erübrigt sich die Verwendung von Wunddrainagen im ambulanten Sektor weitestgehend. *„Wunddrainagen sind im ambulanten postoperativen Verlauf kompliziert und unbequem für die Patienten“* (Tab. 6).

Die freien Antworten der Teilnehmer auf die Frage, warum sie keine Drainagen verwenden, reflektieren dieses Feld recht eindeutig (Tab. 2), zeigen aber auch den individuellen generellen Stellenwert von Wunddrainagen unabhängig von inhaltlicher Evidenz oder definierten Indikationen (*„industriegesponserter Mist“, „… aufwendig und ohne wirklichen Nutzen“*) und unterstreichen die Wertigkeit der klinischen Erfahrung und Meinung des einzelnen Operateurs.

Auch die Handhabung der Verweildauer von Drainagen ist individuell recht unterschiedlich und reicht von 1 Tag bis zu >4 Tagen mit breit gefächerten, intuitiven Begründungen: *„individuell“, „bei Sistieren der Sekretion“, „abhängig von der Operation und von der Lokalisation der Wundtiefe u.* *a.“*. Definierte Angaben zur genauen Sekretmenge oder wie lange die Sekretion sistieren sollte, wurden nicht genannt. Dies und auch die Tatsache, dass die Verweildauer der Drainage auch mit vermehrtem Auftreten von Komplikationen verbunden sein kann, ist durchaus diskutabel, da zu dieser Thematik zumindest eine gewisse wissenschaftliche Evidenz besteht: Untersuchungen hinsichtlich der zu definierenden Fördermenge, ab der eine Drainage entfernt wird, wurden bereits vor 10 Jahren von Amir et al. durchgeführt an einem der Dermatochirurgie zumindest recht nahestehenden Gebiet: der Kopf-Hals-Chirurgie. Sie konnten an 43 Patienten zeigen, dass in ihrer Kohorte die größte Exsudatmenge innerhalb der ersten 8 h nach Operation zu beobachten ist, und führten als Entscheidungskriterium zur Entfernung einer Drainage eine Drainagerate von 1 ml/h innerhalb von 8 h (also meist deutlich früher als die bisherige Angabe von <25 ml/Tag) ohne vermehrte Komplikationsrate ein [10].

Willemen et al. konnten zeigen, dass ab einer Liegedauer der Drainage von 48 h eine gesteigerte Keimkontamination der Drainagespitze beobachtet werden kann und somit ab einer Liegedauer von 48 h mit einer erhöhten Gefahr von Wundinfektionen zu rechnen sei; des Weiteren wird die überwiegende Menge von Exsudat auch in dieser Arbeit innerhalb der ersten 24 h gefördert, sodass zusammenfassend kein Benefit in einer Drainagedauer von länger als 24 h zu liegen scheint [11]. Insbesondere lange Verweildauern von >4 Tagen erscheinen kontraproduktiv hinsichtlich einer sich möglicherweise entwickelnden Wundinfektion. Wiederum stellt die reine Infektionsprophylaxe keine Indikation zur Verwendung einer Wunddrainage dar [12] Ergänzend muss diskutiert werden, ob nicht längere Verweildauern eine prolongierte Wundsekretion induzieren durch die Stimulation einer Gewebereaktion bzw. Erzeugung eines Sogs auf das Gewebe [10].

Komplikationen wurden von jedem zweiten Teilnehmer der Studie beobachtet, wobei analog zu Simgen et al. Zeichen der Wundinfektion (Schwellung, Rötung, laborchemische Infektparameter) genannt wurden und auch Lymphfisteln von den Teilnehmern beobachtet wurden. Entgegen den Erfahrungen von Simgen et al. spielen Schmerzen in der hiesigen Umfrage eine recht große Rolle und werden als führende Komplikation von Wunddrainagen genannt [9]. Interessant, aber in sich konsistent ist daher das Vorgehen von 16,3 % der Teilnehmer, generell bei Verwendung von Drainagen auch eine postoperative Antibiotikaprophylaxe durchzuführen.

Erwähnenswert ist auch die beobachtete Altersabhängigkeit der Verwendung von Drainagen durch die Teilnehmer der Umfrage: Generell verwenden jüngere Kollegen häufiger Drainagen, wobei größere Operationen bei jüngeren Kollegen eher zur Anwendung einer Drainage motivieren. Möglich wäre, dass die Verwendung von Wunddrainagen überwiegend durch jüngere Kollegen präferiert wird, um gewisse alters- und erfahrungsbedingte Unsicherheiten in der Versorgung operativer Patienten auszugleichen, da ein (scheinbares) Gefühl der Sicherheit erzeugt wird (Schutz vor Seromen, Schutz vor Nachblutungen etc.). Dieses Phänomen, dass eine noch nicht ausgereifte chirurgische Technik zu einer überdurchschnittlich hohen Rate an Verwendung der Wunddrainagen führen kann, beschrieben bereits Willy et al. Sie postulierten auch, dass es möglicherweise besser wäre, in solchen Fällen gar keine Wunddrainage zu verwenden [13].

Dieser Faktor des ureigenen Wunsches nach größtmöglicher Sicherheit auch im eigenen operativen Tun scheint sich mit höherem Alter und steigendem Erfahrungsgrad der Kollegen auszugleichen:* „mehr Erfahrung, wann ein Verzicht möglich ist“ *oder* „schlechte Erfahrung …“* (Tab. 6), bzw. mit der Erfahrung, dass auch die Operationsdauer eine Rolle bei der Entstehung von Wundinfektionen spielt, die ihrerseits durch Drainagen gehäuft beobachtet wird (die Operationsdauer spielt in jüngeren Jahren keine große Rolle), zu relativieren. Die Rate an Komplikationen nach operativen Eingriffen steigt mit wachsendem Body-Mass-Index und Nikotinabusus sowie sinkendem Allgemeinzustand/steigenden Komorbiditäten an [14]; dies wurde auch in der vorliegenden Studie durch die Teilnehmer als Erfahrungswert so geäußert.

Zusammenfassend verwendet die Mehrzahl der Teilnehmer Wunddrainagen und dies mehrheitlich intuitiv. Allgemeine Anwendungsgewohnheiten scheinen augenfällig vom Alter und operativen Erfahrungsgrad der Teilnehmer abzuhängen, von der Frage, wo die Teilnehmer arbeiten (Klinik vs. Praxis), sowie von vielfältigen patienteneigenen Faktoren: Adipositas, Raucherstatus, Größe der Operation und Komplexität des operativen Eingriffes. Einheitliche fixe evidenzbasierte Parameter rund um die Verwendung von Wunddrainagen existieren allerdings nicht. Daher gilt nach wie vor für diese Thematik, den Erfahrungsschatz von langjährig operativ tätigen Kollegen zu nutzen und zu schätzen, ähnlich der Einschätzung von Garcia-Elias et al. Sie stellten fest, dass auch in ihrem eigenen Wirkungsbereich die evidenzbasierte Medizin keinen großen Einfluss auf ihr chirurgisches Wirken hatte [15].

Das rezente Verlangen nach harten evidenzbasierten Entscheidungen ist in der Dermatochirurgie im Allgemeinen und hinsichtlich von Wunddrainagen im Speziellen aktuell nicht uneingeschränkt möglich, da diese Evidenzen fehlen, eine hoch qualitative Therapie jedoch trotzdem möglich ist. Heterogene Fallsituationen und unterschiedliche chirurgische Schulen erschweren Vergleiche und Bewertungen verschiedener Methoden und Handlungsweisen erheblich. Ähnlich wie die Handchirurgie ist die Dermatochirurgie ein Fach, in der es außerordentlich schwierig ist, Studien und damit Evidenz zu etablieren [15]: Große Fallzahlen von Patienten mit gleichen Diagnosen an gleichen Lokalisationen von gleicher bis ähnlicher Größe zu identifizieren, die von unterschiedlichen Dermatochirurgen mit vergleichbarem Erfahrungsgrad und dermatochirurgischer Schule durch gleiche Operations- und Rekonstruktionstechniken operiert werden, ist wünschenswert, aber fern jeder Realität.

Somit wird auch bei der Beurteilung der Notwendigkeit einer Wunddrainage vorerst weiterhin ein individuell unterschiedlich ausgeprägtes Sicherheitsbedürfnis bei den einen und „eminenzbasiertes“ Handeln bei den anderen Dermatochirurgen eine große Rolle spielen. Dies ist vor dem Hintergrund einer dringend notwendigen Balance zwischen evidenzassistierter Exzellenz und dem Instinkt des erfahrenen Seniorkollegen wesentlich und kann für unsere dermatochirurgischen Patienten sowie unsere Subspezialisierung eigentlich nur von Benefit sein.

## Fazit für die Praxis

- Die Verwendung von Drainagesystemen in der Dermatochirurgie erfolgt bislang ohne evidenzbasierte Daten.
- Es wurde eine internetbasierte Umfrage unter den Mitgliedern der DGDC e. V. (Deutsche Gesellschaft für Dermatochirurgie e. V.) zur Verwendung von Drainagesystemen durchgeführt.
- Die Mehrzahl der Teilnehmer verwenden Wunddrainagen und dies mehrheitlich intuitiv.
- Bei der Beurteilung der Notwendigkeit einer Wunddrainage scheint ein individuell unterschiedlich ausgeprägtes Sicherheitsbedürfnis bei den einen und „eminenzbasiertes“ Handeln bei den anderen Dermatochirurgen eine große Rolle zu spielen.
